# Female-Specific Flightless (fsRIDL) Phenotype for Control of *Aedes albopictus*

**DOI:** 10.1371/journal.pntd.0001724

**Published:** 2012-07-10

**Authors:** Geneviève M. C. Labbé, Sarah Scaife, Siân A. Morgan, Zoë H. Curtis, Luke Alphey

**Affiliations:** 1 Oxitec Limited, Oxford, United Kingdom; 2 Division of Biology, Imperial College London Silwood Park, Ascot, United Kingdom; 3 Department of Zoology, University of Oxford, Oxford, United Kingdom; Liverpool School of Tropical Medicine, United Kingdom

## Abstract

**Background:**

*Aedes albopictus*, the Asian tiger mosquito, is a vector of several arboviruses including dengue and chikungunya, and is also a significant nuisance mosquito. It is one of the most invasive of mosquitoes with a relentlessly increasing geographic distribution. Conventional control methods have so far failed to control *Ae. albopictus* adequately. Novel genetics-based strategies offer a promising alternative or aid towards efficient control of this mosquito.

**Methodology/Principal Findings:**

We describe here the isolation, characterisation and use of the *Ae. albopictus Actin-4* gene to drive a dominant lethal gene in the indirect flight muscles of *Ae. albopictus*, thus inducing a conditional female-specific late-acting flightless phenotype. We also show that in this context, the *Actin-4* regulatory regions from both *Ae. albopictus* and *Ae. aegypti* can be used to provide conditional female-specific flightlessness in either species.

**Conclusion/Significance:**

With the disease-transmitting females incapacitated, the female flightless phenotype encompasses a genetic sexing mechanism and would be suitable for controlling *Ae. albopictus* using a male-only release approach as part of an integrated pest management strategy.

## Author Summary

The Asian Tiger mosquito, *Aedes albopictus*, is a highly invasive species which took advantage of human activity to spread from South-East Asia to Africa, North and South America, and Europe in the past forty years. Beyond the annoying biting nuisance, this mosquito is also a significant public health threat, capable of transmitting dengue and responsible for an important chikungunya outbreak in the Indian Ocean in 2007. This mosquito is notoriously difficult to control using current methods, but control techniques involving the release of genetically sterile males have recently shown promising results against the closely related yellow fever mosquito, *Aedes aegypti*. Wild females inseminated by sterile males have non-viable progeny so if enough sterile males are released, the target population crashes. Female mosquitoes, even if sterile, would bite and potentially acquire and transmit pathogens, so it is crucial to minimise the release of such females. Here we describe the development of genetically engineered strains of the Asian Tiger mosquito to use in such control programmes: the females are unable to fly unless reared with an antidote, thus facilitating male-only releases. The daughters of released males will also be flightless, a lethal phenotype in the field, thus reducing the population and preventing disease transmission.

## Introduction

The Asian tiger mosquito, *Aedes albopictus* (Skuse), is a vector of several arboviruses including dengue and chikungunya. This highly invasive species originating from Southeast Asia has travelled the world in the last forty years and is now established in Europe, North and South America, Africa, the Middle East and the Caribbean. In the absence of a vaccine or antiviral drugs, efficient mosquito control strategies are crucial. Novel control methods are being developed that involve the use of genetically modified mosquitoes to either suppress the target population or replace it with a pathogen-resistant strain [1], [2], [3], [4], [5], [6].

The sterile insect technique (SIT) is a pest population control method developed in the 1950s which relies on releasing large numbers of males sterilised by irradiation to compete for mates with the wild-type, consequently reducing the proportion of viable offspring [7], [8]. Despite large-scale success against some agricultural pest insects, and some promising successes of this technique against mosquitoes in the 1970s, application of the SIT to mosquito control consistently suffered from the lack of an efficient sexing system in order to eliminate disease-transmitting females before releasing the sterile males [9]. In addition, the irradiation process can impose a significant fitness cost on mosquito species [10]. Furthermore, mathematical modelling shows that the early embryonic lethality caused by the irradiation of paternal sperm is sub-optimal as it reduces the number of immature mosquitoes competing for resources during the density-dependent larval stages [11], [12], [13].

The RIDL system [14] is a variant of SIT which replaces irradiation by genetically engineered inducible sterilisation, an approach offering more flexibility with regards to the time of death, the sex and even the tissues targeted by the sterilising mechanism. Initial estimates suggest that this approach may provide an attractive alternative or complement to additional control methods [3], [13], [15]. In the RIDL systems so far developed, sterility is induced by conditional zygotic expression of a dominant lethal gene: the tetracycline-repressed transactivator tTA [16] is placed under the control of a suitable promoter, while a lethal gene is placed under the control of the tTA response element *tet*O. In the absence of tetracycline, the tTA transactivator binds to tetO and, via activation of a suitable minimal promoter, induces expression of the dominant lethal gene. Tetracycline prevents tTA from binding to the tetO sites, thereby repressing the system and allowing RIDL insects to develop normally on a diet supplemented with tetracycline. However, in the wild, progeny of released RIDL insects will express the lethal gene and consequently die.

The development of a late-acting RIDL strain of *Ae. aegypti* was reported in 2007 [11]. For *Ae. aegypti*, efficient physical sex-separation systems based on pupal size are available; this has allowed male-only release of this strain in successful field trials [17], [18]. A genetics-based alternative would eliminate this labour-intensive step, and is the only option for the wide range of insects, including most mosquitoes, for which reliable physical sex-separation methods are not available. Female-specific (fsRIDL) has additional potential advantages in terms of resistance management that could be highly advantageous in the context of an integrated pest management programme [19]. Genetic sexing RIDL strains of the Mediterranean fruit fly, *Ceratitis capitata*, have been produced based on the sex-specific splicing properties of the *tra* gene [20], but no *tra* homologue has yet been found in mosquitoes.

The *Ae. aegypti Actin-4* gene (*AeAct-4*) is specific to the indirect flight muscles of females with expression starting in L4 larvae [21]: an ideal combination of both female-specificity and late-acting expression allowing production of genetic sexing RIDL strains with a post-larval lethality. Fu *et al.* recently reported the development of a RIDL strain exploiting the promoter and sex-specific alternative splicing of the *AeAct-4* gene and exhibiting a repressible female flightless phenotype [22]. Inability to fly incapacitates females at almost the latest possible stage in development prior to biting. Wise de Valdez *et al.* showed that periodic release of this strain could eliminate cage populations of *Ae. aegypti* [23]. This phenotype is indirectly lethal to females; ability to fly is essential in the field to access sugar resources and escape predation. Flight ability is also needed in both lab and field for mating, as well as – in the field – to acquire a blood meal, so flightless mosquitoes are functionally sterile. A female-flightless phenotype would therefore also permit the release of eggs directly into artificial or pre-existing breeding sites, from which RIDL males would emerge to seek wild females.

With a view to applying the same type of genetic control to populations of *Ae. albopictus* as that proposed by Fu *et al.* [22], we have isolated and characterised a segment of the *Ae. albopictus Actin-4* (*AealbAct-4*) gene. *AealbAct-4* showed functional and sequence similarities to its *Ae. aegypti* homologue: RIDL strains of both *Ae. albopictus* and *Ae. aegypti* carrying a construct based on the *AealbAct-4* promoter and 5′UTR displayed a repressible female flightless phenotype, as did an *Ae. albopictus* strain carrying an *AeAct-4*-based construct. These results indicate that the *Actin-4* promoters from *Ae. aegypti* and *Ae. albopictus* are substantially interchangeable for transgenic-based RIDL strategies in these species.

## Materials and Methods

### Strains background, rearing and transformation

The *Ae. albopictus* and *Ae. aegypti* wild-type strains originated from Malaysia and were colonised by the Institute of Medical Research (Kuala Lumpur) in 2006 and 1977, respectively. The insectary was kept at 27°C (±1°C) and 70% (±10%) relative humidity. Larvae were fed on crushed dry fish food (TetraMin flake food from Tetra GmbH, Germany) and adults on 10% glucose supplemented with 14 µg/ml penicillin and 14 µg/ml streptomycin. Females were fed on horse blood using a *Hemotek* Insect Feeding System (Discovery Workshops, Accrington, UK) set at 37°C.

Pre-blastoderm embryos were prepared for injection and micro-injected as described [24]. Injection mixtures consisted of 300 or 350 ng/µl of donor plasmid (OX3688 and OX4358, respectively), 300 ng/µl of *piggyBac* mRNA [24] and 30 µg/ml of chlortetracycline in injection buffer (5 mM KCl and 0.1 mM NaH_2_PO_4_, pH 6.8). phsp-pBac helper plasmid [25] was also included in the OX3688 mixture to a final concentration of 200 ng/µl as previously described [24]. Injected G_0_ adults were crossed in pools (males in pools of 2 for 24 hours then merged in pools of 24; females in pools of 100) to wild-type counterparts. G_1_ larvae were screened for fluorescence using a Leica MZ95 microscope with the appropriate filter sets from Chroma Technology (Rockingham, VT) (filters: AmCyan: exciter D436/20×; emitter D480/40 m; DsRed2: exciter HQ545/30×; emitter HQ620/60 m). Transgenic lines were established from single G_1_ positive adults. Lines named with different letters have founders from different G_0_ pools and are therefore independent genomic integrations. Lines derived from the same G_0_ pool were characterised and flanking sequences used to confirm their independence from each other (data not shown). Only lines showing a 1∶1 fluorescent to wild-type ratio in the progeny of heterozygous to wild-type crosses, consistent with single transgene insertion, were kept for phenotype analysis (data not shown).

Pictures of fluorescent larvae were taken with a Canon PowerShot S5IS camera with an MM99 adaptor (Martin microscopes) to fit into the eyepiece.

### Isolation of the *Aedes albopictus Actin-4* gene

*Ae. aegypti Actin-4* (*AeAct-4*, AY531222), *Ae. aegypti Actin-3* (*AeAct-3*, AY289765) and *Anopheles gambiae Actin-1* (*AnAct-1*, XM315270, which we considered from sequence analysis likely to be the *An. gambiae* homologue of *Ae. aegypti Actin-4*) sequences were aligned using ClustalW (EBI). Primers AeA4F1 and AeA4R2 were designed in regions which were conserved between *AeAct-4* and *AnAct-1* but differed from *AeAct-3*, and used to amplify *Ae. albopictus* wild-type genomic DNA. The resulting PCR product was cloned and sequenced. BLAST alignment confirmed strong sequence similarity to *Ae. aegypti Actin-4*. This sequence was extended by a combination of 5′RACE and PCR techniques. 5′RACE was performed using the Ambion FirstChoice RLM-RACE kit according to the manufacturer's instructions, using primers AlbA4Race and AlbA4RaceN on 7 µg total RNA extracted from 2 pooled female pupae; adaptor-mediated PCR on genomic DNA was used to extend the sequence from the beginning of the 5′UTR back into the promoter region and from the exon 1 and 2 sequences to obtain the intron sequence. Comparison of cDNA and gDNA sequences revealed a large intron in the 5′UTR. 745 bp upstream from the start of the 5′UTR, a coding sequence with BLAST homology to *Ae. aegypti* sensory neuron membrane protein 2 was found, delimiting the maximum promoter fragment unless the genes overlap.

### Plasmid construction

The OX3688 construct is identical to the OX3604 plasmid ([22], JN936856), apart from a correction: the 3×P3-DsRed marker cassette at one end of OX3604 was subsequently found to be 3×P3-AmCyan instead. This was corrected by exchanging a *PacI*-*SpeI* cassette to make OX3688, which therefore represents the structure originally intended for OX3604. Note that OX3604 encodes a tTA-like protein, tTAV [11]; relative to plasmid OX513 (formerly LA513, [11]) the tTAV coding region in OX3604 has altered nucleotide sequence which we now refer to as tTAV2. The complete sequence of the OX3604 transposon has been deposited in GenBank with accession number JN936856.

OX4358 construction: A start codon was engineered in the *AealbAct-4* gene's 5′ UTR 43 bp before the 5′ donor site of the intron by PCR. Two PCR products, promoter-intron and intron-truncated exon 2, were amplified from wild-type *Ae. albopictus* genomic DNA using primer pairs AlbA4proAscF-AlbA4intSpeR and AlbA4intSpeF-AlbA4ex2BglR. The two PCR products were ligated at the *Spe*l site; the ligated product was cloned in front of the fusion gene ubiquitin–tTAV2–K10 3′UTR previously constructed [22]. The engineered start codon was in frame with the tTAV2 coding sequence. This gene cassette was inserted into an existing *piggyBac* construct, containing the Hr5-IE1 enhancer-promoter from the baculovirus *Autographa californica* MNPV [26] driving AmCyan (Clontech).

### RT-PCR

In order to study the endogenous *Actin-4* gene from *Ae. albopictus*, RNA was extracted from pooled samples of three wild-type male pupae and two wild-type female pupae, using Tri Reagent (Ambion), according to the manufacturer's instructions. RNA samples were treated with DNAse I (Roche) and quantified on a Pharmacia Biotech GeneQuant II RNA/DNA calculator. One-step RT-PCR was carried out on 200 ng RNA using SuperScript III One-step RT-PCR System with Platinum *Taq* DNA Polymerase (Invitrogen) and primers in the 5′UTR (AlbA4UTRF) and in exon 2 (AlbA4FlR) (see Table S1), according to the kit protocol. PCR conditions were 50°C for 30 min, 94°C for 2 min followed by 40 cycles of 94°C for 15 s, 55°C for 30 s and 68°C for 1.5 min, with a final elongation at 68°C for 5 min.

RT-PCR was carried out on male and female pupae of OX4358 and OX3688 individuals as above, using primers AlbA4BsmF and UbiR2 for OX4358, and Aeact4-ex1, Aeact4-ex1′ and Diag2-ubi for OX3688 (Table S1), and the same PCR conditions, to confirm that sex-specific splicing was occurring as predicted in this context. Amplified fragments were verified further by sequencing (GATC Biotech, Konstanz, Germany) following gel extraction and purification using the Qiaquick gel extraction kit (Qiagen), according to manufacturer's instructions.

### Phenotype analysis

For phenotypic analysis of the transgenic lines, eggs were hatched on day 1. On day 2, “on tet” and “off tet” trays (11×19 cm bottom surface) were set up with 300 heterozygous larvae in 300 ml of pure water (1 larva/ml), respectively with or without a supplement of chlortetracycline hydrochloride (Sigma-Aldrich, Gillingham, UK) to a final concentration of 30 µg/ml. Water was changed on days 6 and 11. Larvae were fed crushed dry fish food (TetraMin flake food from Tetra GmbH, Melle, Germany): 12 mg/tray on days 2, 3 and 17; 24 mg/tray on days 4, 9, 11 and 12; 48 mg/tray on day 5; 96 mg/tray on days 6, 7 and 8.

Sexes were separated as pupae and placed in cages into 5×5×5 cm weighing boats. Emerged adults were separated each day and their flying ability evaluated the following day by aspirating out flying individuals while tapping the cage to stimulate immobile adults. Flying adults were recorded as a proportion of pupae placed in the cage as some death - due to incomplete eclosion or drowning soon after eclosion - occurred before flying ability could be established. The proportion of pupae producing flying adults was therefore used as the measure of fitness in the present studies. For reference, the wild-type strain reared off tetracycline has an eclosion rate (± SE) of 93.83% (±0.98%) for males and 91.17% (±1.82%) for females.

## Results

### Isolation and characterisation of the *Aedes albopictus Actin-4* gene

**Figure 1:**
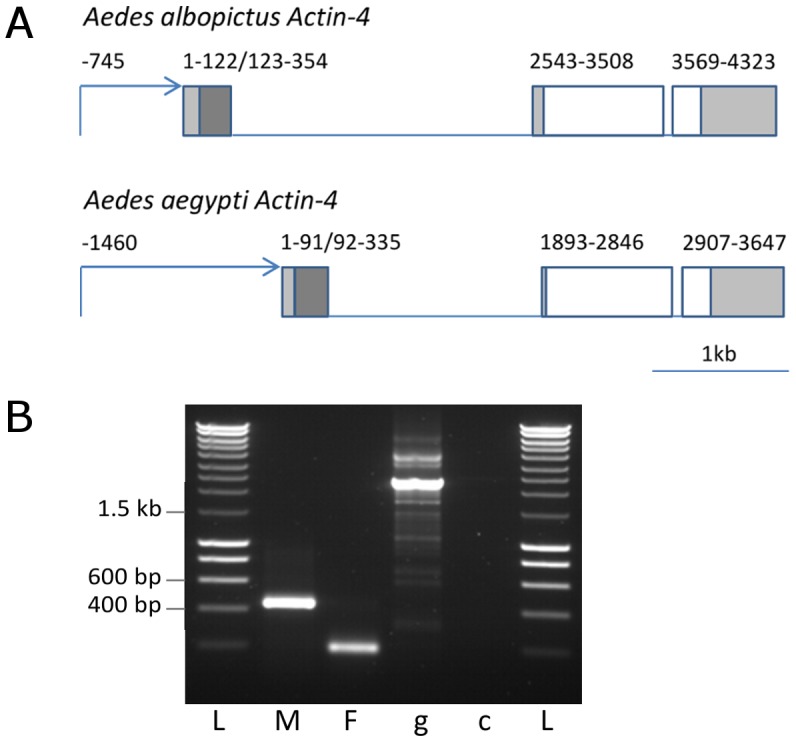
Characterisation of *Ae. albopictus Actin-4* gene. A: Gene structure of *Aedes albopictus* and *Aedes aegypti Actin-4*. Putative promoters are indicated by arrows, exons are shown as boxes, introns as lines. Non-coding 5′ and 3′UTR are shaded pale grey; the male-specific exons are shaded dark grey. B: RT-PCR confirming differential splicing in male (M) and female (F) *Ae. albopictus* pupae; genomic (g) and no template control (c) are also shown. L: DNA size marker (Smartladder, Eurogentec). Sizes of the major bands are consistent with the predicted gene structure (panel A and Table S1); sequence of the cDNA bands confirmed splice sites (not shown).

The *Ae. albopictus Actin-4* gene (*AealbAct-4*) was isolated as described in Materials and Methods. The gDNA and cDNA sequences have been deposited in GenBank (Accession numbers: JN709493 and JN709492, respectively). The sequence showed high conservation with *AeAct-4* (and also to *AgAct-1*, not shown), particularly in the coding sequence (Figure S1). The positions of the introns are conserved, as is the gene structure with respect to sex-specific splicing (Figure 1A). The sex-specific splicing was confirmed by RT-PCR (Figure 1B).

### Conditional female flightlessness in *Ae. aegypti* and *Ae. albopictus* using *Ae. albopictus Actin-4*

**Figure 2:**
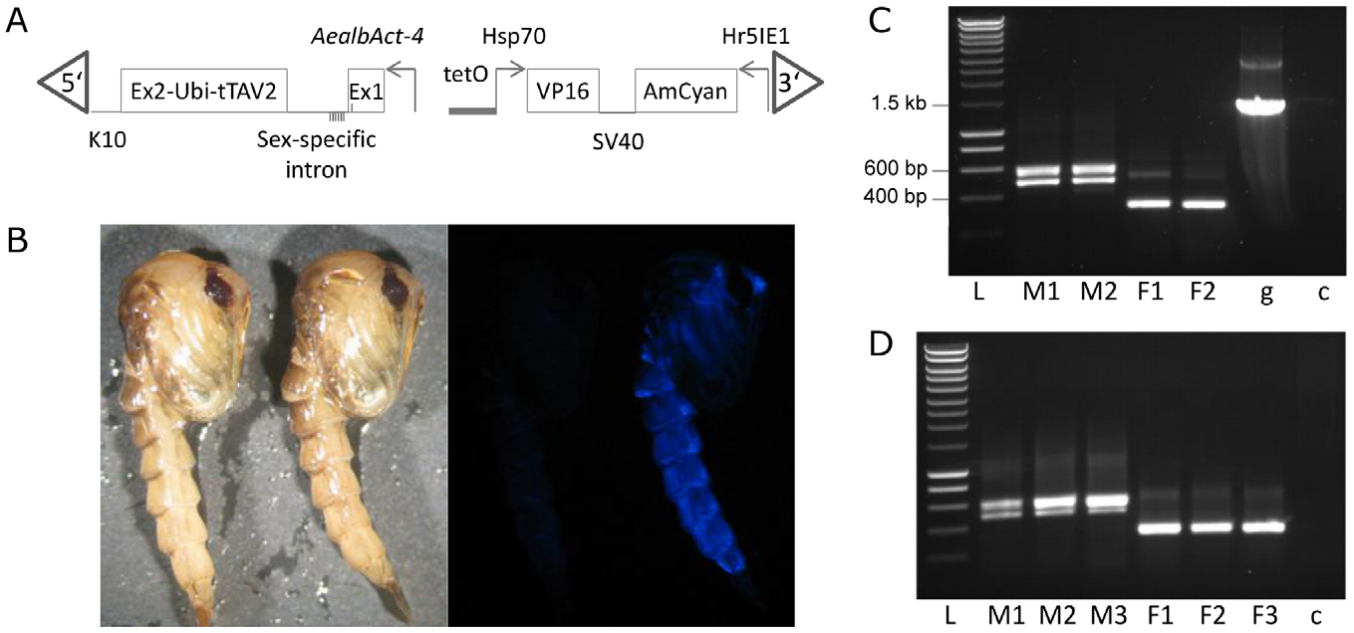
OX4358 structure, fluorescence phenotype and splicing. A: Map of the OX4358 construct. Promoters are indicated by arrows, exons are shown as boxes, introns as horizontal lines. The engineered start codon is indicated by a bar in the *Actin-4* exon 1 (Ex1), whilst stop codons in the male exon are shown by bars below the line. B: Phenotype of wild-type (left) and OX4358F1-Aal (right) pupae under white light (left panel) and blue filter (right panel). C: RT-PCR analysis of transcripts from *Ae. albopictus* OX4358, from two male and two female pupae (M1, M2, F1 and F2, respectively), gDNA amplification (g) and no-template control (c). L: DNA size marker (Smartladder, Eurogentec). D: RT-PCR analysis of transcripts from *Ae. aegypti* OX4358, from three male and three female pupae (M1, M2, M3, F1, F2, and F3 respectively) and no template control (c). Sequencing of the PCR products revealed that splicing occurs as in the native gene, except for a second male-specific transcript in which exon 1 has an extra 75 bp in both *Ae. aegypti* and *Ae. albopictus* (see text).

The *AealbAct-4* promoter and 5′UTR (containing the alternatively spliced region) were used to make construct OX4358 (Figure 2A). OX4358 includes a Hr5IE1-AmCyan-SV40 marker gene leading to strong expression of the AmCyan fluorescent protein all over the body at every developmental stage and allowing simple and reliable screening of the transgenics (Figure 2B). The *Ae. albopictus Actin-4* (*AealbAct-4*) promoter was placed in front of the *AealbAct-4* exon 1, in which a start codon has been engineered. The *AealbAct-4* sex-specific intron was shortened by internal deletion but preserving the male-specific transcript which provides multiple stop codons (Figure 2A, bars below the intron line). The *AealbAct-4* exon 2 was cloned in frame with tTAV2, a variant of tTA optimised for expression in insects (JN936856). Expression of VP16 is activated by the binding of tTAV2; this occurs only where tTAV2 is expressed and in the absence of tetracycline. Transgenic lines carrying OX4358 were obtained for both *Ae. albopictus* and *Ae. aegypti*.

RT-PCR analysis of OX4358 transgenic individuals confirmed sex-specific splicing in both *Ae. albopictus* and *Ae. aegypti* (Figure 2C and 2D, respectively). Sequencing of the PCR products revealed that splicing occurs as in the native gene, except for a second male-specific transcript in which exon 1 has an extra 75 bp. This extra transcript may be a result of truncation of the intron and/or exon 2, disrupting splicing. However, it still leads to a frame-shift between the start codon and the tTAV2 coding sequence, as with the canonical splice variant, and should not interfere with the intended function of the construct.

*Ae. aegypti* and *Ae. albopictus* transgenic lines carrying the OX4358 construct were reared on and off tetracycline and their flying/non-flying phenotype assessed. *piggyBac*–based transgenes insert at any of a very large number of sites, therefore each transgene is embedded in a different chromatin context, which may influence its expression and associated phenotype [27]. A spectrum of phenotypes was therefore anticipated. Of the 20 independent lines obtained in *Ae. albopictus*, four exhibited a non-repressible flightless females phenotype, giving essentially no flying females when reared on tetracycline; five lines were found to be male-linked; these nine lines were not analysed further. The remaining eleven lines were tested on and off tetracycline (Table 1); eight had a repressible female flightless phenotype, with no females flying off tetracycline and between 22 and 55% of females flying on tetracycline. Three lines showed no obvious sex-specific flightless phenotype. Eleven independent lines were obtained in *Ae. aegypti*, including two male-linked insertions. The other nine lines were tested on and off tetracycline (Table 1); four had a repressible female flightless phenotype, with no flying females off tetracycline and 57 to 96% females flying on tetracycline. Four lines showed incomplete penetrance, with 1 to 43% females able to fly off tetracycline. The last line showed no clear flightless phenotype, with 93% females flying off tetracycline while 84% females flew on tetracycline. No impairment was observed in the ability of males to fly when reared off tetracycline. In fact there was generally a slightly higher percentage of flying males when reared off tetracycline compared to rearing on tetracycline (Table 1).

**Table 1:** Phenotype of OX4358 transgenic lines.

Species	Line	Females ON		Females OFF		Males ON		Males OFF	
*Ae. aegypti*	A2	94%	(n = 159)	1%	(n = 209)	99%	(n = 184)	95%	(n = 240)
	A3	57%	(n = 79)	0%	(n = 74)	100%	(n = 49)	100%	(n = 68)
	B3	96%	(n = 124)	0%	(n = 147)	98%	(n = 228)	96%	(n = 207)
	C2	83%	(n = 77)	43%	(n = 77)	92%	(n = 101)	95%	(n = 75)
	D7	89%	(n = 64)	0%	(n = 64)	95%	(n = 62)	96%	(n = 81)
	E4	94%	(n = 72)	0%	(n = 82)	93%	(n = 82)	93%	(n = 74)
	E8	95%	(n = 83)	13%	(n = 63)	86%	(n = 65)	91%	(n = 86)
	F4	84%	(n = 56)	93%	(n = 70)	90%	(n = 94)	92%	(n = 76)
	H5	88%	(n = 97)	13%	(n = 87)	91%	(n = 106)	76%	(n = 144)
	WT	99%	(n = 487)	99%	(n = 454)	99%	(n = 536)	99%	(n = 462)
*Ae. albopictus*	A6	22%	(n = 194)	0%	(n = 211)	41%	(n = 234)	52%	(n = 244)
	A7	29%	(n = 174)	0%	(n = 78)	55%	(n = 186)	60%	(n = 108)
	B1	46%	(n = 82)	0%	(n = 65)	74%	(n = 90)	71%	(n = 77)
	B2	73%	(n = 172)	73.%	(n = 182)	62%	(n = 161)	66%	(n = 166)
	C4	36%	(n = 247)	0%	(n = 234)	33%	(n = 334)	68%	(n = 262)
	D2	50%	(n = 80)	68%	(n = 19)	63%	(n = 81)	65%	(n = 34)
	E5	55%	(n = 55)	0%	(n = 23)	76%	(n = 75)	80%	(n = 25)
	F1	54%	(n = 69)	0%	(n = 64)	47%	(n = 89)	52%	(n = 86)
	F5	67%	(n = 18)	100%	(n = 2)	79%	(n = 19)	89%	(n = 9)
	H2	51%	(n = 55)	0%	(n = 17)	50%	(n = 90)	46%	(n = 24)
	I1	53%	(n = 129)	0%	(n = 74)	51%	(n = 147)	53%	(n = 104)
	WT	82%	(n = 229)	83%	(n = 241)	71%	(n = 252)	80%	(n = 246)

Flying ability of transgenic OX4358 lines of *Ae. aegypti* and *Ae. albopictus* was recorded after being reared with (ON) or without (OFF) a supplement of the tetracycline antidote. The percentage of each sex found to be capable of flying is indicated, calculated as a proportion of the number of pupae (n) placed in each cage.

### Conditional female flightlessness in *Ae. albopictus* using *Ae. aegypti Actin-4*

**Figure 3:**
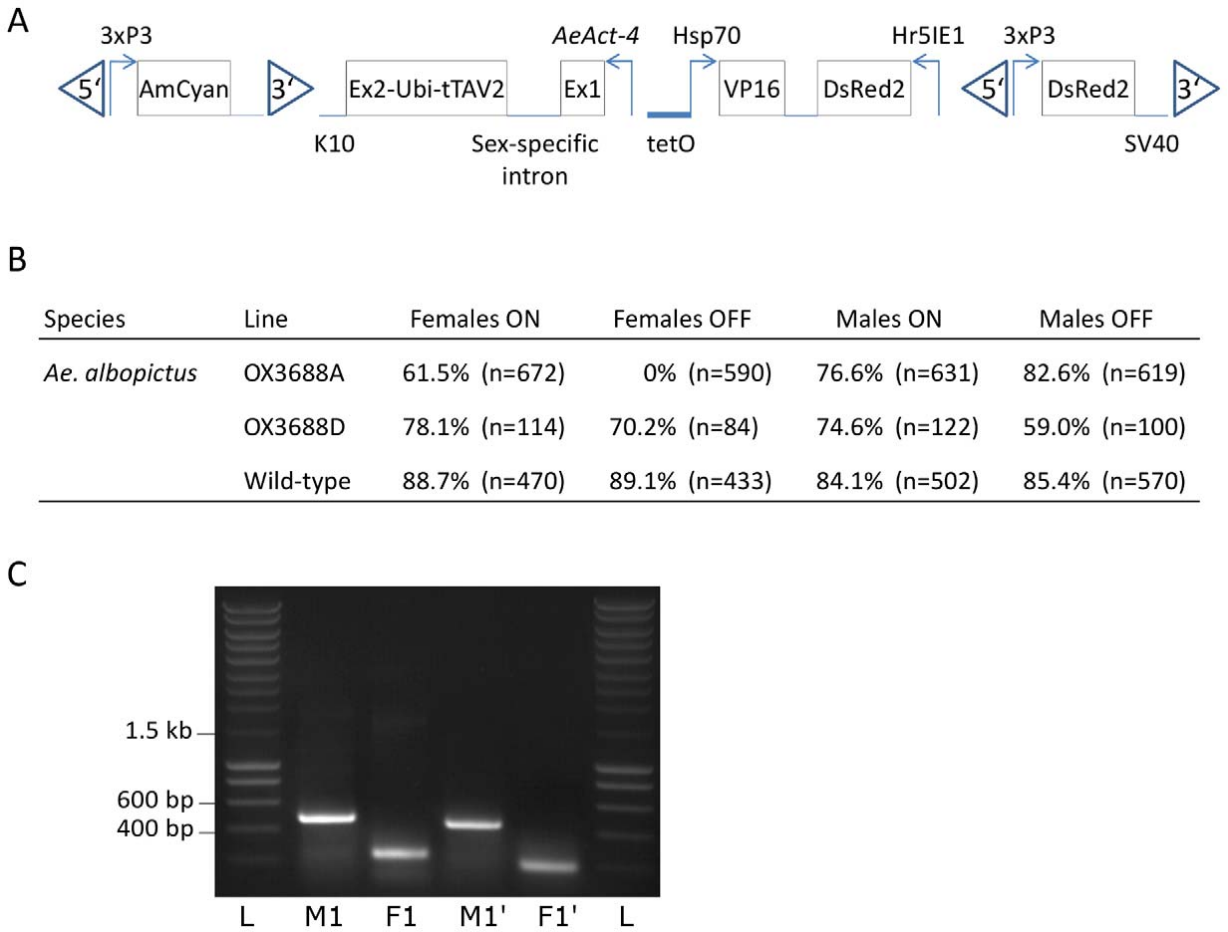
OX3688 construct and phenotypes. A: Map of the OX3688 construct. Promoters are indicated by arrows, exons are shown as boxes, introns as horizontal lines. B: Flying phenotype of transgenic heterozygous OX3688A and D lines of *Ae. albopictus* after being reared with (ON) or without (OFF) tetracycline antidote. Pupae were screened to separate wild-type from transgenics, sexed, and numbers recorded (n). The percentage of flying adults was calculated as a proportion of the number of pupae. C: RT-PCR on male (M) and female (F) *Ae. albopictus* OX3688A-Aal pupae showing differential sex-specific splicing of the construct. RT-PCR was performed using primer pairs Diag2-ubi and Aeact4-ex1 (M1 and F1) and Diag2-ubi and Aeact4-ex1′ (M1′ and F1′) in ubiquitin and *AeAct-4* exon 1. Ladder (L): DNA size marker (Smartladder, Eurogentec). Band sizes are consistent with predictions (Table S1).

The development of a tetracycline-repressible female flightless phenotype in *Ae. aegypti* has recently been reported using the *AeAct-4* promoter [22]. We transformed *Ae. albopictus* with a similar construct, OX3688 (Figure 3A). Three transgenic lines were produced. In one of them the flightless phenotype was not repressed by tetracycline at the concentrations used, producing flightless females even when reared on tetracycline. Line OX3688A-Aal showed a repressible female-specific flightless phenotype, with females flying on tetracycline but flightless off tetracycline, and males flying irrespective of tetracycline (Figure 3B, Video S1, Video S2). Line OX3688D-Aal did not show female-specific flightlessness off tetracycline (Figure 3B). RT-PCR was performed on OX3688A-Aal male and female pupae, finding sex-specific splicing consistent with the pattern seen in the native gene (Figure 3C). Sequencing of the RT-PCR fragments indicated that the splicing occurred just as in *Ae. aegypti* (data not shown).

## Discussion

**Figure 4:**
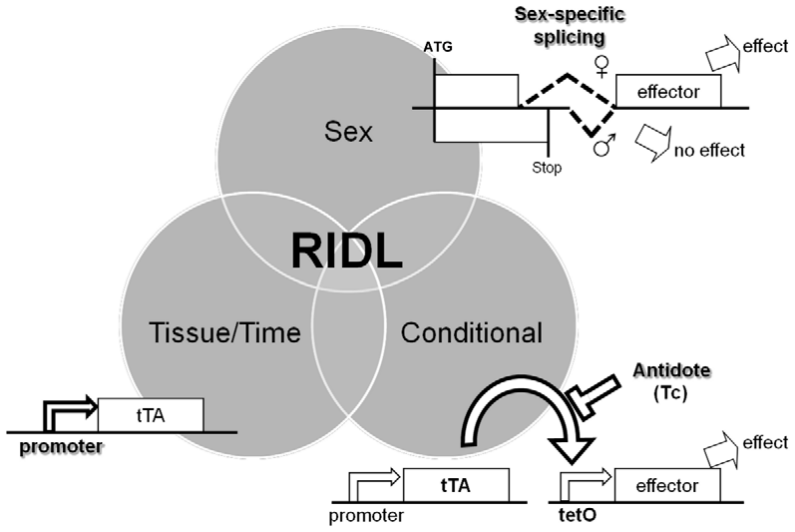
RIDL as applied synthetic biology: combinatorial control of gene expression with defined modular control elements. In the RIDL constructs described here, the promoter, sex-specific intron and tetracycline-repressible trans-activation mechanism (tTA or alternatives bind and activate the tetO enhancer in the absence of tetracycline Tc) act as independent control elements; logic gates which combine specific inputs (respectively Tissue/Time, Sex, Conditionality) to give predetermined logical outputs.

Fu *et al.* recently reported the engineering of a conditional female flightless phenotype in *Ae. aegypti* using a DNA segment from the *Ae. aegypti Actin-4* gene (*AeAct-4*) [22]. The results presented in this paper show that this segment retains its key properties in *Ae. albopictus* and can be used to generate a similar phenotype. Moreover, replacing the *AeAct-4* sequence with one from its *Ae. albopictus* homologue also induced a conditional female flightless phenotype in both *Ae. albopictus* and *Ae. aegypti*. This is the first report of an engineered *Ae. albopictus* phenotype which could be used successfully for population control of the species. The promoter, sex-specific intron and tTAV2 act as independent control elements; logic gates which combine specific inputs (tissue, sex, tetracycline) to give predetermined logical outputs (Figure 4). Such applied synthetic biology of pest insects is in its infancy, but already real-world applications can be seen.

Although some sequence variation between the two homologous genes were observed, the two promoters and the sex-specific splicing appear to function similarly in both species. This suggests that they may also behave similarly in other closely related species. The availability of two elements of similar function but different sequence might also be advantageous if female-specific expression of two genes is required simultaneously as it would limit homologous recombinations within the construct.

In spite of the general similarity of the different promoters in the two species, Ae. *albopictus* appeared more affected by the OX4358 RIDL constructs than *Ae. aegypti*, with relatively low percentages of flying females on tetracycline and males both on and off tetracycline compared to *Ae. aegypti* lines carrying the same construct. Several potential explanations can be proposed to explain this apparent difference between *Ae. aegypti* and *Ae. albopictus*. The *Actin-4* promoters may express at a higher level in *Ae. albopictus* (or, equivalently, the mRNA or protein might be more stable, or the mRNA translated more efficiently), leading to a higher production of tTAV2; *Ae. albopictus* indirect flight muscles (IFMs) may be more sensitive to the over-expression of VP16 than *Ae. aegypti* IFMs, although the apparent effect on males may also indicate a somewhat less tight regulation of the *Actin-4* promoter in *Ae. albopictus*; or tetracycline may be metabolised slightly differently in the two species leading to sub-optimal repression of the tetO-VP16 in *Ae. albopictus*. Wide phenotypic variations were also noted among the OX4358-Aal lines, while the *Ae. aegypti* lines generally had percentages of flying females on tetracycline (except line A3) and of flying males both on and off tetracycline that were more similar to wild-type. One might have expected the OX4358 construct to be more tightly controlled in *Ae. albopictus*, as it is based on the *Ae. albopictus Actin-4* gene. On the other hand, the *Ae. aegypti* wild-type strain also displayed greater fitness in relation to eclosion rates/ability to fly than the *Ae. albopictus* wild-type strain, so the observed differences may relate in part to the much longer colonisation time of the *Ae. aegypti* strain which may have led to a more lab-adapted and homogeneous genetic background. Such heterogeneity in the *Ae. albopictus* background, despite reducing the rearing efficiency of the strains in a laboratory environment, may represent an advantage in the field as males may be more apt to survive and find mates than some more lab-adapted counterparts.

We have shown that it is possible to engineer late-acting, repressible female-specific transgene expression to provide a conditional female-specific flightless phenotype in *Ae. albopictus*. This study represents a significant step towards genetic control of *Ae. albopictus*. The flightless phenotype appeared to be somewhat less tightly regulated in *Ae. albopictus* than in *Ae. aegypti*; this may lead to lower mass production efficiency and male competitiveness and thereby affect the economics of a control programme based on this technology, or at least these prototype strains. Further work will be required to develop and characterise *Ae. albopictus* strains homozygous for these transgenes and assess their suitability and effectiveness in suppressing wild populations. Such new methods are needed. As recent chikingunya outbreaks in the Pacific Ocean [28], [29] highlight, the public health threat posed by the spread of *Ae. albopictus*, though less than that of *Ae. aegypti*, remains significant and cannot be considered to be adequately controlled by currently available methods.

## Supporting Information

Figure S1 **Clustal alignment of** ***Aedes aegypti*** **and** ***Aedes albopictus Actin-4*** **cDNA sequences (AeAct-4 and AealbAct-4, respectively).** Positions of introns are marked with a vertical line, translation start and stop are underlined, the male-specific exon is shown in italics.
(TIF)

Table S1 **Primer sequences and expected product sizes.**
(DOC)

Video S1 **OX3688A-Aal adult males reared in the absence of tetracycline are able to fly normally.**
(WMV)

Video S2 **OX3688A-Aal adult females reared in the absence of tetracycline are unable to fly.**
(WMV)
