# Supplementary figures and images for: Female-Specific Flightless (fsRIDL) Phenotype for Control of Aedes albopictus
Source: PLoS Negl Trop Dis. 2012 Jul 10;6(7):e1724. doi: 10.1371/journal.pntd.0001724 (PMC3393675; doi:10.1371/journal.pntd.0001724)

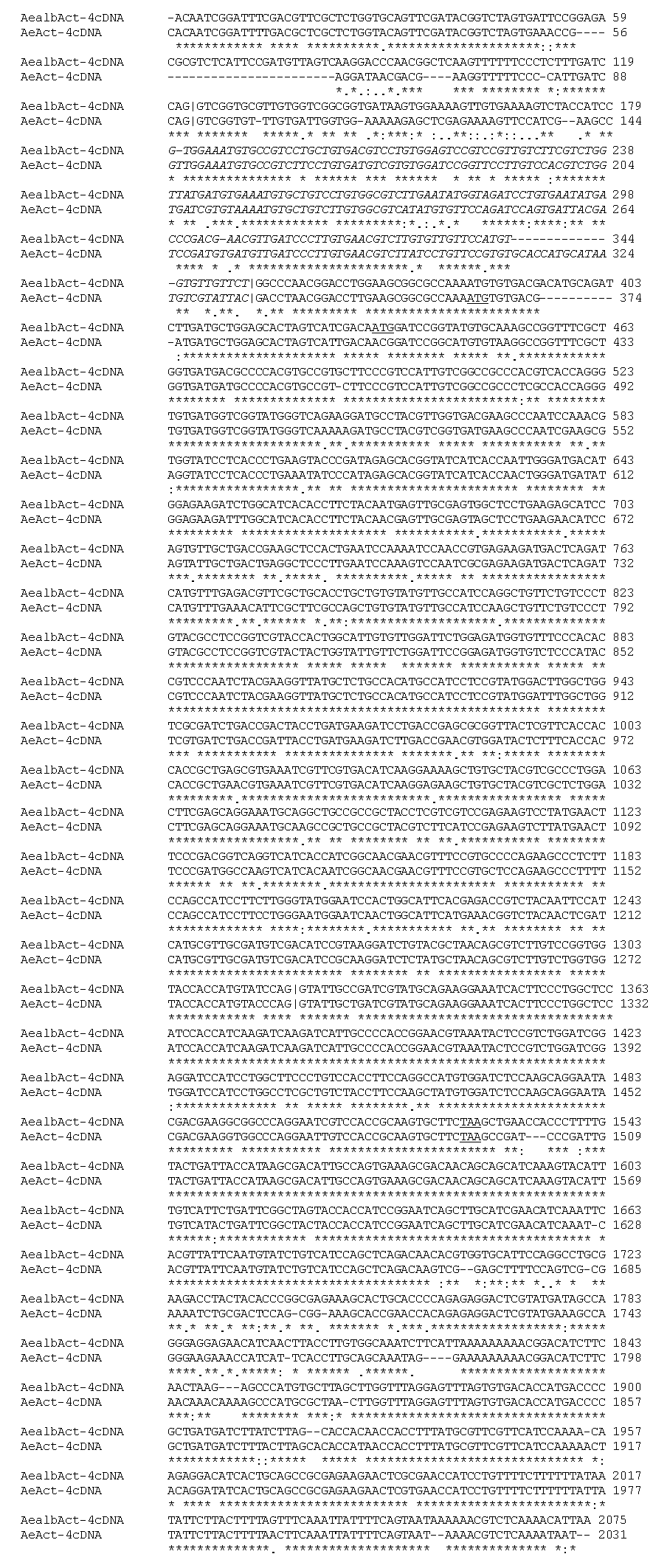

Supplement: Figure S1 — Clustal alignment of Aedes aegypti and Aedes albopictus Actin-4 cDNA sequences (AeAct-4 and AealbAct-4, respectively). Positions of introns are marked with a vertical line, translation start and stop are underlined, the male-specific exon is shown in italics. (TIF) [file pntd.0001724.s001.tif]
