# Supplementary material for: Female-Specific Flightless (fsRIDL) Phenotype for Control of Aedes albopictus
Source: PLoS Negl Trop Dis. 2012 Jul 10;6(7):e1724. doi: 10.1371/journal.pntd.0001724 (PMC3393675; doi:10.1371/journal.pntd.0001724)
Supplement: Table S1 — Primer sequences and expected product sizes. (DOC) [file pntd.0001724.s002.doc]

| F primer name | F primer sequence | R primer name | R primer sequence | Product size (bp) |
| --- | --- | --- | --- | --- |
| AeA4F1 | GTGTGACGATGATGCTGGAGCACTAG | AeA4R2 | CTGGGTACATGGTGGTACCACCAGAC | 928 |
| AlbA4Race | GGGATTCAGTGGAGCTTCGGTCAGCAGG |  |  |  |
| AlbA4RaceN | TGGATTGGGCTTCGTCACCAACGTAG |  |  |  |
| AlbA4proAscF | GGTGTGGGCGCGCCTGATCGGTAAGGTAAGTAAGCATCCGAG | AlbA4intSpeR | GGTGTGACTAGTGTTGACAACTCTTCTAGTTTCTCGGCC | 1566 |
| AlbA4intSpeF | GGTGTGACTAGTCCAAAATGAACGTGGACCAGCC | AlbA4ex2BglR | GGTGTGAGATCTGCATGTCGTCACACATTTTGGCGCCGCTTCCAGGTCCGTTGGGTCC | 579 |
| AlbA4UTRF | GTTAGTCAAGGACCCAACGGCTC | AlbA4FlR | CACACCCTGGTGACGTGGGC | 452, 220, 2640 |
| AlbA4BsmF | GGTGTGCGTCTCACCACCATGTTAGTCAAGGACCCAACGGCTCAAG | UbiR2 | cataccaccgcgcaggcg | 569, 337, 1526 |
| Diag2-ubi | GGATGCCCTCCTTGTCCTGG | Aeact4-ex1 | CAATCGGATTTTGACGCTCGCT | 495, 252, 2051 |
| Diag2-ubi | GGATGCCCTCCTTGTCCTGG | Aeact4-ex1’ | CATGGAAACCGAGGATAACGACGA | 446, 203, 2002 |
